# Cannabis for the Management of Cancer Symptoms: THC Version 2.0?

**DOI:** 10.1089/can.2018.0009

**Published:** 2018-05-01

**Authors:** Manuel Guzmán

**Affiliations:** Department of Biochemistry and Molecular Biology, CIBERNED, IUIN and IRYCIS, Complutense University, Madrid, Spain.

The landscape of medical cannabis is rapidly expanding. Cannabis preparations have been used in medicine for millennia, and now there is a strong renaissance in the study of their therapeutic properties. The vast majority of controlled clinical trials that support the medical use of what is commonly known as “cannabis” or “marijuana” have actually been conducted with purified cannabinoids or a single extract of *Cannabis sativa* that contains an equimolecular proportion of Δ^9^-THC and CBD. Based on these studies, THC/dronabinol (Marinol) and its synthetic analogue nabilone (Cesamet), as well as nabiximols (Sativex), are already approved by several regulatory agencies, including FDA, Health Canada, and EMA, as antiemetic, anticachexic, analgesic, or antispastic medicines.

However, crude cannabis preparations remain by far the most frequent source of cannabinoids for patients worldwide. Medical cannabis dispensation programs have already been implemented in more than half of the states in the United States, as well as in a growing number of countries globally. Although this “living laboratory” of medical cannabis users has indeed the potential of providing a treasure of observational data, unfortunately very few studies have examined the therapeutic value of, for example, cannabis oils or vaporized herbal cannabis. In fact, basically nothing is known on the demographic and pathological characteristics of patients using cannabis, the patterns of use of different cannabis strains, the efficacy and safety profiles of those cannabis preparations, and their most appropriate doses and routes of administration.

Israel, led by Prof. Raphael Mechoulam, is one of the most prominent countries in scientific and clinical cannabis research worldwide. On these grounds, and since 2007, the Israeli Ministry of Health has been providing authorizations for medical cannabis use. Nowadays there are >30,000 patients in Israel taking cannabis, especially for the palliation of cancer symptoms. In the March 2018 issue of *European Journal of Internal Medicine*, Bar-Lev Schleider et al. provide a valuable epidemiological insight into ∼3000 of those cancer patients who had been prescribed cannabis for managing their malignancy-associated symptoms.^1^ This cohort, although somewhat heterogeneous, could be generally considered as “seriously ill.” For example, at baseline, about half of the patients suffered from stage 4 cancers; about half of the patients reported their pain as intense (8 over 10); the average age of the patients was relatively advanced (∼60-year-old); and the number of symptoms reported per patient was very high (an average of 11, including sleep problems, pain, anxiety and depression, weakness and fatigue, nausea and vomiting, and appetite loss). After 6 months of follow-up, 24.9% of patients had died and 18.8% had stopped the treatment. Of the remaining, 60.6% responded to the questionnaires. Regarding overall efficacy, 95.9% of patients reported an (either significant or moderate) improvement in their condition. Likewise, only 18.8% of patients reported good/very good quality of life before treatment initiation, whereas 69.5% did so after the 6-month cannabis regime. Regarding overall safety, cannabis was generally well tolerated, and most side effects reported, such as dizziness, dry mouth, and somnolence, could be considered as mild, especially in the context of an advanced cancer patient population.

This study provides a precious piece of information on the use of medical cannabis for the management of cancer symptoms. The sample population was large, and a prospective follow-up with rather high adherence and response rates was achieved. However, as inherent to most observational studies, the study is also bound to recognizable limitations such as the absence of a placebo group, the patient's self-selection bias, the lack of control over other concomitant treatments (e.g., chemotherapy), and the use of subjective questionnaires rather than objective parameters to assess the patient's health status.

Perhaps the observation of the study that has been most actively stressed by the mass media (see, e.g., https://www.rollingstone.com/culture/news/new-study-suggests-pot-could-help-end-opioid-dependency-w517178) is the observed drop in opioid use: although opioids were the most consumed drug by patients at enrollment, after 6 months of cannabis use, 36.0% of them had stopped taking opioids entirely, and an additional 9.9% decreased their dosage. In the face of the dramatic opioid epidemic in the United States, this finding offers reasons for hope. However, in my opinion, this potential inverse relationship between cannabis and opioid use in the clinical setting deserves at least a cautionary comment. For example, data from Medicare Part D enrollees from 2010 to 2013 revealed that prescriptions for pain medication, including opioids for pain, fell in states with medical cannabis laws, resulting in savings of US$165.2 million in 2013 alone.^2^ Similarly, a 2015 RAND Bing Center for Health Economics study found that states permitting medical cannabis dispensaries experienced a relative decrease in opioid addictions and opioid overdose deaths.^3^ Another recent report, by collecting survey data from medical cannabis patients, also suggested that cannabis contributes to reducing opioid-based pain medication.^4^ Clinical research into the interactions between cannabis and opioids in pain patients is limited, but pilot studies indicate that medical cannabis intake tends to reduce the dose of opioids required for pain relief.^5,6^ The website of the National Institute on Drug Abuse, one of the National Institutes of Health (https://www.drugabuse.gov/publications/marijuana/marijuana-safe-effective-medicine), upon reviewing of this current evidence, notes that “Though none of these studies are definitive, they cumulatively suggest that medical marijuana products may have a role in reducing the use of opioids needed to control pain.”

The notion that opioid use decreases upon concomitant cannabinoid use is actually challenging, and stands supported by some pre-clinical and “real-patient” data, but, unfortunately, it is not sustained yet by rigorously controlled clinical studies. We cannot forget that association is not causality, and that the plural of anecdote is not evidence. Identifying effective pain-management strategies alternative to opioid analgesics is a clear public health priority. As Bar-Lev Schleider et al. discuss in their article, most patients using medical cannabis report that it has fewer and less severe side effects than their concurrent prescription drugs, especially opioids. Hence, well-designed, large controlled trials are urgently warranted to determine whether combining cannabinoids with opioids can actually reduce the amount of opioids necessary to manage pain.

*C. sativa* is a plant with a complex and varied chemical composition,^7^ so it is essential to define its precise chemotypes to offer one of them to a specific patient as a valid therapeutic option. Considering the hundreds of compounds that are present in cannabis (cannabinoids, terpenes, polyphenols, steroids, flavonoids, etc.), it is clear that a substantial part of them can, at least theoretically, exert biodynamic actions on the human body. In the study by Bar-Lev Schleider et al., each patient was prescribed at least one out of 18 different cannabis strains. Most patients used THC-rich, CBD-poor, *indica*-like strains, which were usually complemented with various *sativa*-like and/or CBD-rich strains. Moreover, almost half of the patients combined the use of oils and inflorescences (including flowers, capsules, and cigarettes). Unfortunately, the study had not sufficient statistical robustness to substratify the patients for this complex array of cannabis preparations.

In any case, based on our current knowledge on the mechanism of cannabinoid action, it is most conceivable that the therapeutic activity of these THC-rich preparations for treating classical cancer symptoms is due to the THC-induced activation of cannabinoid CB_1_ receptors located on precise anatomical sites.^8,9^ This includes, for example, inhibition of nausea and vomiting, stimulation of appetite, attenuation of cachexia/energy expenditure, and reduction of pain, in which effects mediated by cannabinoid CB_2_ receptors could also be involved. Likewise, engagement of cannabinoid CB_1_ receptors located on specific brain areas most likely underlies the typical side effects described in the commented study, such as dizziness, somnolence, confusion, and disorientation.

Beyond THC, it is becoming increasingly accepted that CBD, aside from exerting its own therapeutic activity, buffers the psychoactive risk of cannabis.^10^ Thus, THC/CBD-balanced preparations, obviously if well produced and standardized, could be considered a therapeutically safer option than dronabinol or nabilone, whose therapeutic windows are usually very narrow. Other constituents of cannabis, especially terpenes (e.g., myrcene, α-pinene, and β-caryophyllene), have been proposed to exert synergic therapeutic actions with phytocannabinoids.^11^ However, scientific proof for this potential “entourage effect” is still missing.

In sum, the one million dollar question of “what would be the best cannabis chemotype and, in particular, THC/CBD ratio for each particular patient in each particular pathological status?” remains a pending question in the field. It seems really far from our logistic, economic, and human resources (especially under the current restrictive frameworks that regulate clinical research with substances considered to be narcotic drugs) to conceive clinical trials with tens of thousands of patients to compare different pure cannabinoids, either alone or in combination, with other cannabinoids and terpenes, versus different cannabis extracts, all of them administered by different routes and for different diseases. Therefore, more realistically, a multifactorial approach to the problem could be considered from three complementary levels: (1) Pre-clinical studies aimed at evaluating interactions between different (cannabinoid and noncannabinoid) compounds from a biochemical, pharmacological, and behavioral perspective could suggest candidate combinations to be used in therapy. (2) Controlled clinical trials with the most appropriate selection of such combinations of compounds could provide accurate data on efficacy and safety (e.g., dosage and treatment durations, and pharmacokinetic parameters). (3) Observational studies with different chemotypes, preparations, and delivery procedures, mainly under the umbrella of medicinal cannabis dispensation programs, could provide signs of whether, for example, cannabis oils or herbal cannabis are more effective and/or better tolerated than THC and CBD (alone or combined in different proportions).

Compared with pure cannabinoids, medical-grade cannabis preparations may offer, in theory, the possibility to personalize—and therefore improve—therapeutic interventions in terms of different THC/CBD ratios, activating (*sativa*-like) versus sedating (*indica*-like) cannabis strains, and slow (e.g., oral oils) versus fast (e.g., vaporized inflorescences) routes of delivery. However, as already mentioned, additional rigorous clinical studies are warranted for us to move from scattered anecdotal evidence to clinical knowledge. Nowadays, on practical grounds, interpretation of empirical records on medical cannabis use, combined with a rational application of our current understanding of the mechanism of cannabinoid action, as well as some “trial and error,” may be the only way to delineate which cannabis preparations may adjust best (in terms of efficacy and tolerability) to the specific needs of each patient at each disease stage. Hopefully, this relatively fragile strategy will evolve in the near future for the appreciable benefit of the patient.

## Abbreviations Used

CBD: cannabidiol
THC: tetrahydrocannabinol
